# The importance of rehabilitation concerning upper extremity amputees: A Systematic review

**DOI:** 10.12669/pjms.325.9922

**Published:** 2016

**Authors:** Kardem Soyer, Banu Unver, Seval Tamer, Ozlem Ulger

**Affiliations:** 1 Kardem Soyer, MSc PT. Department of Physiotherapy and Rehabilitation, Hacettepe University Faculty of Health Science, Ankara, Turkey; 2 Banu Unver, MSc PT. Department of Physiotherapy and Rehabilitation, Istanbul Aydin University Faculty of Health Science, Istanbul, Turkey; 3 Seval Tamer, MSc PT. Department of Physiotherapy and Rehabilitation, Hacettepe University Faculty of Health Science, Ankara, Turkey; 4 Ozlem Ulger, PT. Associate Professor, Department of Physiotherapy and Rehabilitation, Hacettepe University Faculty of Health Science, Ankara, Turkey

**Keywords:** Upper limb, Prosthetics, Amputee Rehabilitation

## Abstract

**Objective:**

To evaluate and point out the importance of prosthetic rehabilitation of upper extremity.

**Methods:**

A systematic literature search was performed to identify studies concerning prosthetic rehabilitation in upper extremity. The PRISMA Statement 2009 was used to establish the study and the methodological quality was assessed.

**Results:**

The literature search identified 620 studies. Of these 620, 9 studies fulfilled the inclusion criteria and were included for data extraction. The studies pointed out the upper limb prosthetic rehabilitation protocols consist of general exercise programme, motor tasks, phantom exercises, Muscle Training System, edema control, functional activities, signal strengthening, prosthetic education exercises, neuromuscular reeducation, virtual image and virtual reality exercises.

**Conclusions:**

The current systematic literature review has shown that the prosthetic rehabilitation seems promising especially for upper extremity amputees.

## INTRODUCTION

The upper extremity, is one of the most important part of the body that possess functional ability to perform daily activities, self care duties, hobbies and sports. Depending on fine motor properties of this extremity effection of functions and activities are inevitable for the individuals. New technology and materials have been advanced in prosthetic designs to activate people who trust to artificial limbs to achieve feats and new advancements in the sensorial and motor control restoration with targeted reinnervation and hand transplantation never dreamed before.^1^,^2^ Prosthetic training is necessary for learning how to use and associate the prosthesis into daily life. Effective and adequate rehabilitation studies, not only improve individual’s functionality and satisfaction but also increase self care independence which provide the success of prosthetic design.^3^

In the literature studies have focused on the effects of rehabilitation approaches; physiotherapy methods like strengthening exercises and functional activities, virtual reality and mirror therapy.^3^,^4^ The conclusions of the studies show that prosthetic rehabilitation facilitates prosthetic usage which leads to increase in independence and improvement in functional capacities.^3^,^4^

The aims of this systematic review was to evaluate and point out the importance of prosthetic rehabilitation of upper extremity and to anticipate to the professionals in this field. For this reason we conducted this study with studies based on upper extremity amputee rehabilitation published within last 10 years.

## METHODS

In this systematic review, The PRISMA Statement 2009 was used to establish the study and we sticked with The PRISMA Checklist and Flow Diagram.^5^

***Literature search:*** The following keywords were used to search the electronical database PubMed and Web of Science (WoS) (2005-2015): upper extremity, upper limb, upper extremity amputee, upper limb amputee, transhumeral and transradial. An additional search was performed using the following keywords: amputation, rehabilitation, training, functional treatment and physical therapy. The search was limited to humans and the search filters for PubMed are clinical trials, controlled clinical trial, journal article, comprehensive study, meta-analysis, randomised controlled trial, review, systematic review and full text. The only search filters for Web of Science (WoS) is full text. The search strategy that was used is presented in the Appendix. Searching the database and the reference lists of appropriate publications were checked.

### Study selection

The studies that were identified using the keywords were independently assessed by three reviewers. The following inclusion criteria were used to include studies for the review:

**Table:** Appendix- PubMed and Web of Science (WoS) search strategy

1.	‘Upper extremity’ [MeSH]
2.	‘Upper limb’ [MeSH]
3.	‘Transhumeral’
4.	‘Transradial’
5.	‘Amputee’
6.	‘Amputation’
7.	‘Rehabilitation’
8.	‘Training’
9.	‘Functional treatment’
10.	‘Physical tderapy’
11.	Nos. 1 and 5 and 7 or 8 or 9 or 10
12.	Nos. 2 and 5 and 7 or 8 or 9 or 10
13.	Nos. 3 and 5 or 6 and 7 or 8 or 9 or 10
14.	Nos. 4 and 5 or 6 and 7 or 8 or 9 or 10

- The study had to related to amputee rehabilitation.
- The study had to be focused on the upper extremity
- The study had to be a full text article in a peer-reviewed journal.

The reviewers decided the studies that should be included in the final review in a consensus meeting. To allow the most complete aspect of the current literature, the search was not limited by publication type or by patient group.

### Methodological judgment

Jovell & Navarro-Rubio’s classification of study designs was used to assess the methodological quality of the articles (Table-I).^6^

**Table-I:** Classification of the study design as described by Jovell & Navarro-Rubio.^6^ This classification was used to assess the methodological quality of the included papers.

*Level*	*Strength of evidence*	*Type of study design*
I	Good	Meta-analysis of randomised controlled trials
II		Large-sample randomised controlled trials
III	Good to fair	Small-sample randomised controlled trials
IV		Nonrandomised controlled prospective trials
V		Nonrandomised controlled retrospective trials
VI	Fair	Cohort studies
VII		Case-control studies
VIII	Poor	Noncontrolled clinical series; descriptive studies
IX		Anecdotes or case reports

### Data analysis

The included articles were reviewed according to a structured diagram. The content of the papers were scanned for: the diagnosis, subjects (n, age, sex), side of lesion, time since injury, design classification, baseline measurements, intervention, outcome measurements and conclusions by the three reviewers. The data of these categories were displayed in the tables. Differences in opinion were analyzed in discussion.

## RESULTS

### Study selection

**Fig.1:**
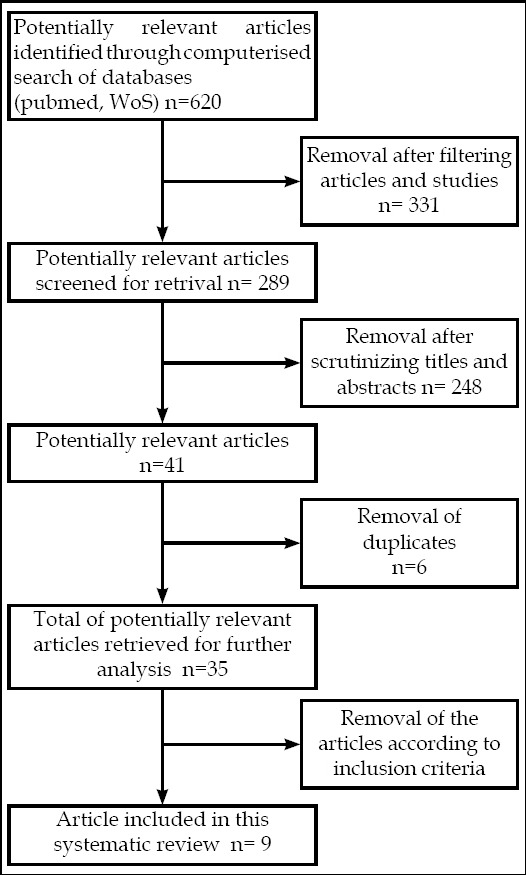
Flow diagram of article retrival and analysis.

The literature search identified 620 studies. Of these 620, 9 studies fulfilled the inclusion criteria and were included for data extraction. Fig.1 All of these 9 studies were involved rehabilitation and included upper limb amputees. Table-II lists the subject characteristics of the studies. The studies included a total of 150 subjects; 116 with upper limb amputation, 11 with lower limb amputation, 6 with brachial plexus avulsion and 17 with orthopaedic upper limb disability. Three of these studies were case reports,^4^,^7^-^8^ 5 of them were noncontrolled clinical series^9^-^12^ and one of them was randomised controlled trial.^13^ Methodological quality-based on the classification of Jovell & Navarro-Rubio^6^ of included studies are listed in Table-III.

**Table-II:** Subject characteristics of the 9 included studies.

*Authors*	*Diagnosis*	*Subjects (n, age in years mean±s.d. (range)), sex)*	*Side of Lesion*	*Time Since Injury in Years (mean±s.d. (range))*
Mercier et al.^11^	Traumatic upper limb amputation	n=2, Age=54 Age= 40	Left	4
		Sex= Not specified	Right	1
Ulger et al.^13^	Traumatic amputees	Experimental group; n= 10, Age= 41.60±4.17, Sex=Not specified	Not specified	Experimental group; 2.7±0.82 months
		Control group; n= 10, Age= 42.10±4.48, Sex=Not specified		Control group; 3.30±1.15 months
Toledo et al.^7^	Arm amputation above the elbow	n=1, Age=58, Sex=Not specified	Right	Not specified
Jönsson et al.^10^	Upper limb loss	n=37 (10 thumbs, 1 partial hand, 10 transradial and16 transhumeral), Age= Not specified, Sex= Not specified	Not specified	Not specified
Dromerick et al.^8^	Shoulder desarticulation, Above elbow amputation	n=1, Age=15, Sex= male	Bilateral Right shoulder disarticulation, left above elbow	3 years
Yancosek et al.^9^	Orthopaedic upper limb disability Transhumeral Transradial	n=35, Orthopaedic upper limb disability (17) Transhumeral (10) Transradial (8) Age= Not specified, Sex= 2 Female/33 Male	Dominant (6), non-dominant (4) Transhumeral Dominant (3), non-dominant (5) Transradial	Not specified
Korkmaz et al.^12^	Congenital limb loss Acquired	n= 40 (Pediatric amputee), Congenital (10 Above elbow, 10 below elbow) Acquired(10 Above elbow, 10 below elbow) Age= Not specified Sex= 19 Male, 21 Female Case report, Male	Not specified	Congenital= 12.40±3.05 Acquired= 3.25±1.77
Resnik et al.^4^	Forequarter Shoulder disarticulation	n= 7 Shoulder disarticulation (3)	Left Not specified	1 year (cancer history) After amputation
Stubblefield et al.^14^	Transhumeral	Transhumeral (4)		

**Table-III:** Study characteristics of the prosthetic rehabilitation with upper limb amputee.

*Authors*	*Methodological Quality*	*Baseline Measurements*	*Intervention*	*Outcome Measurements*	*Conclusion*
Mercier et al.^11^	Level VIII	Phantom limb pain measurement visual analog scale (VAS) Phantom limb pain with VAS (at least 7)	Before; Medication, neuroma removal, TENS, acupucture After; Presenting virtual image of the lost limb The used motor tasks used were the following: flexion/extension of the elbow, pro-supination of the forearm, flexion/extension of the wrist, opening/closing the hand, abduction/adduction of the fingers, thumb-to-finger opposition, flexion/extension of the thumb, grasping an object (such as glass), precision grip with small objects, and dialing a phone number 2 treatment sessions per week for 8 weeks 30-60 minutes	Relief Post 1 w %65.2 %93.5 Relief Post 4w %61.4 %88.9	Movements are easier, especially at the elbow, but still require intense effort Frequent sensations that the hand is sweating and of muscle soreness Tactile sensations when the hand contacts an object Movements are easier with the feedback but feel like they are performed against resistance Tactile sensations during finger opposition and contact with objects The phantom exercises can be used safely to alleviate phantom limb pain in lower and upper limb amputees
Ulger et al.^13^	Level III RCT		Experimental group; i. subjects were asked in which position they felt the phantom limb ii. they were asked to place the intact limb in the same position as they felt their phantom limb iii. they were asked to move both limbs in the opposite direction iv. they were then asked to return to the starting position again Exercises were repeated 15 times or until the phantom pain disappeared (4 weeks) Control group; General exercises: strengthening, streching, dynamic and isometric exercises based on the level of amputation 10 times twice daily, 4 weeks	Experimetal group; Phantom sensation, VAS Pre-test 8.40±1.08 Post-test 6.30±0.95 Phantom pain, VAS Pre-test 9.20±0.79 Post-test 6.10±0.74 Control group; Phantom sensation, VAS Pre-test 8.50±1.08 Post-test 7.90±0.88 Phantom pain, VAS Pre-test 9.30±0.82 Post-test 7.60±0.52
Toledo et al.^7^	Level IX	EMG Signal Acquisition	Muscle Training System with Visual Feedback Three phases of training At each phase, the patient is asked to carry out three increasing levels of strength. When the patient reached to a specific strength level, he must hold on the contraction for 10 seconds followed by 10 seconds of resting until the three levels are completed Forthe first and second phases, the protocol was performed by using the visual feedback, whereas for the third phase the visual -feedback halted. The records of the three phases were around 30 minutes of effective training for each session. 20 sessions total	The patient tends to stabilize the strength of the contractions, achieving a good evolution through the three training stages. The patient has succeeded in controlling the strength of the contractions according to the trained levels.	The proposed training protocol is adequate for educating the patient with upper limb amputation above the elbow, in order to control myoelectrical prosthesis
Jönsson et al.^10^	Level VIII	Function and Quality of Life (QoL)	S1 and S2 surgeries were done to transhumeral, transradial and thumb amputees. Transhumeral amputation; After surgery, the patient is instructed to perform limited range of motion of the shoulder without pain. Three weeks after the surgery the patient can start to practice internal/external rotation of the shoulder to avoid rotational forces of the distal soft tissues. Strengthening exercises for arms, shoulders, chest and back muscles. First, low weights (50-100g) are applied to the training prosthesis and these are increased each week (50-100 g) until the patient reaches the weight of the final prosthesis. Secondly, the patient performs axial weight loading twice daily.12 weeks after the surgery gentle exercises performed with the prosthesis and these increase in the intensity time. Transradial amputation; Used prosthesis as a support in daily activities Thumb amputation;- Range of motion and edema First three months after surgery the thumb prosthesis used for light activities of daily living	Not Specified	Osseointegration has the potential to change the rehabilitation strategy for selected upper limb amputees and is very important platform for introducing new prosthetic technology, due to stable fixation
Stubblefield et al.^14^	Level VIII	Functional assessment	Signal strengthening, strengthening exercises, muscle relaxation exercises, diagnostic fitting, functional exercises, unilateral and bilateral activity exercises before TMR	Functional assessment	The main subject, forthe patients to whom TMR application was done, is to recognise the main principles of TMR by the group members. Being understand the distribution of peripheral nervous system, characteristics of the surgery and the effects on patients are important factors. TMR effectiveness depends on the relationship between the doctor, prosthetist, occupational therapist and the patient.
Dromerick et al.^8^	Level IX	Functional disability test	Proximal muscle strengthening, prostetic training exercises, neuromuscular reeducation (MyoBoy), therapeutic activities	Jebsen-Taylor Hand Function Test, Box and block text of manuel dexterity, Action Research Arm Test	Despite the increase in movement speed to the experienced and motivated upper limb amputee patient application of new prosthesis caused functional limitation to decrease rapidly. Education, maintained development in functional limitation and movement speed, but there was no differences in movement aligment.
Yancosek et al.^9^	Level VIII	Score Measurement (Occupational outcomes) Going back to occupation, daily activities, functional capacity	Virtual reality (The Firearm Training System) Voice-Sensitive Technology Adaptive sports	Valpar Joule FCE system	CFI as a new facility is described that represents theadvanced levels of independence sought by therecovering SMs and fostered through military rehabilitationmedicine.
Korkmaz et al.^12^	Level VIII	Functional assessment	Scapular and shoulder girdle strengthening, back and abdominal muscle strengthening exercises 2 times per day and 15 repeat Self care activities, table activities, household activities, fine motor skills, general activity exercises for prosthetictraining	Child amputee prostetic Project-functional status inventory (CAPP-FSI) Prostetic upper extremity functional index;(PUFI)	For detailed assessment it is important to assess functional activities with or without prostheses. Daily prosthesis usage duration and experiences of the child is one of the important factors for establishing functional status.
Rasnik et al.^4^	Level IX	Phantom pain (in sitting position) 16 controlled DEKA arm system usage described.	VirtualReality Environment3.5 hours (4 couse day 8 session in total)	Question-answer	Fascilitate Virtual Reality Environment(VRE) usage

### Amputation

Hundred and sixteen subjects with upper limb amputations were included in these studies. Fifty eight of these subjects were above elbow amputees and 49 of them were below elbow amputees. Nine of the subjects’ amputation level weren’t specified.

### Intervention

The study characteristics of the prosthetic rehabilitation protocol are listed in Table-III. Two of the included studies used rehabilitation to treat phantom limb pain;^11^,^13^ one of them used presenting virtual image of the lost limb and performing motor tasks,^11^ the other used phantom exercises and general exercise programme including strengthening, stretching, dynamic and isometric exercises.^3^ One of the included studies used Muscle Training System with visual feedback.^7^ One study used edema prevention, range of motion exercises, strengthening exercises and performing daily living activities for training of osseointegration.^10^ One study used signal strengthening, strengthening exercises, muscle relaxation exercises, diagnostic fitting, functional and activity exercises for training amputees with targeted mucle reinnervation (TMR).^14^ One study used proximal muscle strengthening, prosthetic education exercises, neuromuscular reeducation and therapeutic activities.^8^ Two studies used virtual reality exercises;^6^,^9^ one of them used voice sensitive technology and adaptive sports to compare with the effects of virtual reality exercises.^9^ One study used scapular and shoulder girdle, back and abdominal muscle strengthening exercises, self care activities, table and household activities, motor skills and general activity exercises for training child amputees.^12^

### Outcome measurements

Two of the included studies used visual analog scale (VAS) to assess phantom limb pain.^11^,^13^ One study used EMG signals to measure muscle strength.^7^ Other 6 studies used different functional assessments to evaluate effects of the training.^4^,^8^-^10^,^12^,^14^ Outcome measurement methods of the included studies are listed in Table-III.

## DISCUSSION

In the present review, 9 studies were qualitatively analysed to investigate the effects of prosthetic rehabilitation on functional impairement like activities for daily living, sensory function and pain reduction of the upper limb.

Prosthetic rehabilitation is not in its early stages, but only a small number of studies could be included in this review. The methodological quality of the included studies is variable. The highest methodological score according to Jovell and Navarro-Rubio^6^ (Table-I) of the studies in this review is level III, which corresponds to a good strength of evidence. Only one of the 9 included studies was randomised controlled trials (RCT). The remainder of the studies had a poor strength of evidence and mostly concerned as noncontrolled clinical series, descriptive studies or case reports. The studies on amputation of upper limb other than other parts of the body all had a weak methodological quality; hence, these studies were slightly used to draw conclusions regarding the effectiveness of prosthetic rehabilitation. Our conclusions on the effectiveness of prosthetic rehabilitation, as explained in the following, were drawn using studies with thorough contents and conclusions.^4^,^7^-^14^

Note that all of the studies except one are nonrandomised trials and those studies have a poor methological score relative to the other study, there are aspects of these studies that possibly bias the results. For instance, the number of participants in the studies is small in general. Another factor that may have biased the results was that the contents of the prosthetic training programs. Finally, in some of the studies prosthetic rehabilitation was performed in combination with other forms, which made it difficult to determine which part of the treatment contributed to the reported effects.

The amputation leves are miscellaneous, and in all studies the subject characteristics were well described. As regards the contraindications, phantom limb pain is commonly seen after amputation surgery. Three studies were focused on phantom limb pain and reported a positive effects like “movements are easier”, “increase in tactile sensation”, “aleviate the phantom limb pain” especially in traumatic upper limb amputations.

As regards to the prosthetic rehabilitation programs, importantly, exercises like joint movement in normal range, strengthening, stretching exercises are useful in rehabilitation in order to keep muscular structures in balance, for daily activities and self care activities.

Ulger et al., in their randomised controlled trial study stated that both prospective exercises and dynamic and isometric exercises in prosthetic rehabilitation can be used safely to alleviate phantom limb pain in upper limb amputees.^13^ A possible working mechanism of proprioceptive exercises in upper limb amputee is based on the fact that movement of the unimpaired upper limb is used to improve the motor control of the amputated limb. This bilateral movement suggests a bilateral transfer as an origin of the effects of proprioceptive exercises.

The forced attention should be drawn to functional recovery for the upper limb amputees because functionality is important for the individual in order to carry out their daily lifes. Mercier et al. reported that the motor tasks for upper extremity amputations are critically important in order to perform activities like grasping an object, dialing a phone number etc.^11^ Korkmaz et al. reported that with the aim of scapular-shoulder girdle, back-abdominal strengthening exercises and in addition selfcare, table, household activities are useful for the upper limb amputee children for development of fine motor skills during growth.^12^

Since mirror therapy seemed effective with phantom pain,^15^ it was thought that this therapy combined with therapeutic approaches might also work with upper limb ampute.^7^,^11^ The proposed training protocol is adequate for educating the patient with upper limb amputation in order to control myoelectric prosthesis.^7^

Nowadays, technology is employed to advantage in prosthetic rehabilitation, such as in the literature prosthetic education is done with MyoBoy or DEKA arms and virtual reality.^4^,^8^,^9^ In the studies prosthetic rehabilitation were combined with a motor imaginary program, hence, it could be that it was this combination of motor imaginary and exercises that cause the positive effect.^4^,^8^,^9^

After amputation, one of the alternative treatment protocol is osseo integration. Osseo integration has the potential to change the rehabilitation strategy for upper limb amputees and it is believed that it is very important for introducing new prosthetic technology, due to stable fixation.^10^

Prosthetic rehabilitation seems to be effective for the upper limb amputees. But in the researches the assessment methods were mostly subjective. For this reason, in order to support literature and clinical experiences, the evidence based researches should be done.

All in all, the current systematic literature review has shown that the prosthetic rehabilitation seems promising, especially for upper extremity amputees.
